# Cerebral salt wasting in a patient with myeloproliferative neoplasm

**DOI:** 10.1186/s12883-019-1393-4

**Published:** 2019-07-18

**Authors:** Lea Orlik, Reto Venzin, Thomas Fehr, Karin Hohloch

**Affiliations:** 1 Kantonsspital Graubuenden, Internal medicine, Chur, Switzerland; 2 Kantonsspital Graubuenden, Internal medicine, Department of Nephrology, Loestr. 170, Chur, Switzerland; 3 Kantonsspital Graubuenden, Internal medicine, Department of Hematology and Oncology, Loestr. 170, 7000 Chur, CH Switzerland; 4 Department of Hematology and Oncology, Georg August University, 37072 Goettingen, Germany

**Keywords:** Cerebral salt wasting syndrome, Hyponatremia, Myeloproliferative syndrome

## Abstract

**Background:**

Cerebral salt wasting (CSW) is a rare metabolic disorder with severe hyponatremia and volume depletion usually caused by brain injury like trauma, cerebral lesion, tumor or a cerebral hematoma. The renal function is normal with excretion of very high amounts of sodium in the urine. Diagnosis is made by excluding other reasons for hyponatremia, mainly the syndrome of inappropriate antidiuretic hormone secretion (SIADH).

**Case presentation:**

A 60-year-old patient was admitted to the emergency room with pain in the upper abdomen and visual disturbance two weeks after knee replacement. The patient was confused with severe hematoma at the site of the knee endoprosthesis. Laboratory values showed massive thrombocytosis, leukocytosis, anemia, severe hyponatremia and no evidence of infection. CT scan of the abdomen was inconspicuous. Head MRI showed no ischemia or bleeding, but a mild microangiopathy. A myeloproliferative neoplasm (MPN) was suspected and confirmed by bone marrow biopsy. Cerebral salt wasting syndrome was identified as the cause of severe hyponatremia most likely provoked by cerebral microcirculatory disturbance. The hematoma at the operation site was interpreted as a result of a secondary von Willebrand syndrome (vWS) due to the myeloproliferative neoplasm with massive thrombocytosis. After starting cytoreductive therapy with hydroxycarbamide, thrombocytosis and blood sodium slowly improved along with normalization of his mental condition.

**Conclusion:**

To the best of our knowledge this is the first description of a patient with CSW most likely caused by a microcirculatory disturbance due to a massive thrombocytosis in the context of a myeloproliferative neoplasm.

## Background

Cerebral salt wasting (CSW), first described in 1950 [1], is a rare metabolic disorder defined by hyponatremia and extracellular volume depletion in patients with normal adrenal and thyroid function. CSW often occurs after a cerebral injury or trauma, frequently subarachnoidal hemorrhage, brain surgery or stroke. CSW is also reported in patients with carcinomatous or infectious meningitis, encephalitis, poliomyelitis and central nervous tumors. The supposed pathomechanism in CSW is the release of brain natriuretic peptide (BNP) by the injured brain followed by natriuresis and volume depletion [2]. Alternatively, an alteration of sympathetic renal afferent fibers with an increased production of natriuretic peptides has been proposed as an underlying cause for CWS. Therefore, this syndrome was recently also described as renal salt wasting syndrome (RSW) [3]. The existence and prevalence of CSW/RSW is often debated, and the accuracy of discrimination between the syndrome of inappropriate antidiuretic hormone secretion (SIADH) and CSW/RWS is difficult and only possible by determination of the volume status of the patient. The challenge to assess the exact volume status as the sole clinical criteria to distinguish CSW/RSW and SIADH in clinical practice is the major reason for these difficulties. All other clinical and laboratory findings are overlapping in both syndromes [4, 5]. Therefore existence of RWS/CWS is often put in question mostly due to a lack of defined diagnostic criteria and an uncertainty of the pathophysiological mechanisms. Also more recent publications suggest the syndrome should be named “renal salt wasting” rather than “cerebral salt wasting” with several cases described in the literature in patients without cerebral disease [3]. The clinical course with large saline and volume requirement along with simultaneous renal salt loss confirms the diagnosis. Accuracy of diagnosis is crucial due to strikingly different treatment options in SIADH and CSW/RSW. In SIADH fluid restriction is the standard of care, whereas in CSW/RSW saline infusion is the treatment of choice.

## Case presentation

This is a case report of a 60-year-old male patient, with knee replacement two weeks ago, presented with pain in the upper abdomen and large hematoma around his operated knee. He reported impaired vision in the last two weeks and appeared confused. Clinical examination revealed only slight pain of the upper abdomen. Laboratory results showed severe thrombocytosis (1385 G/l), leukocytosis (49.7 G/l), anemia (98 g/l) and hypoosmolar hyponatremia (105 mmol/l). No clinical or laboratory signs of infection were found. CT scan of thorax and abdomen was inconspicuous. Head MRI showed only a mild microangiopathy with no evidence of hemorrhage or ischemia nor of sinus venous thrombosis. However, a jugular vein thrombosis was detected.

**Fig. 1:**
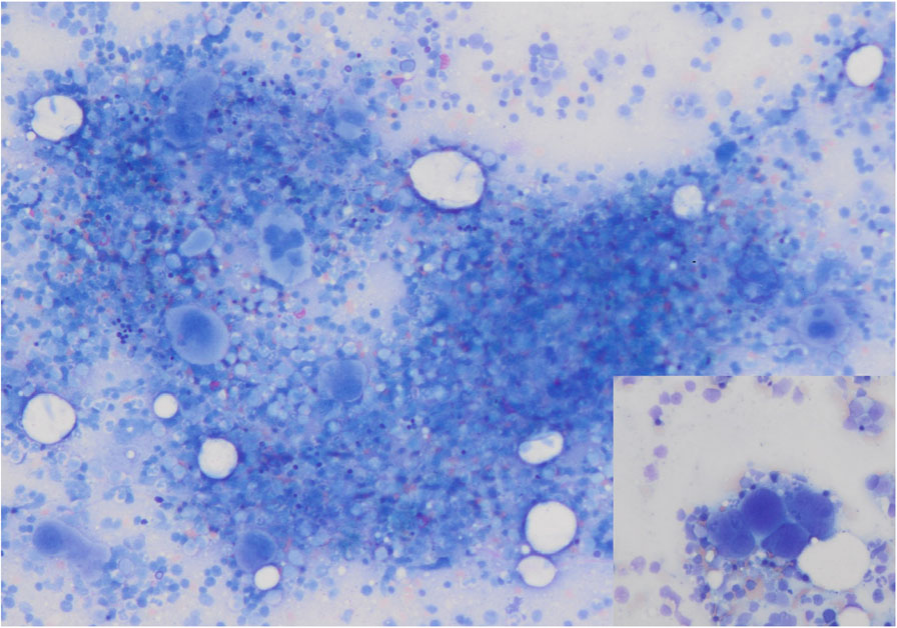
Hypercellular bone marrow at initial diagnosis, Clusters of megakaryocytes (small picture)

Because of excessively high platelet and leukocyte counts and thrombosis, a myeloproliferative neoplasm (MPN) was suspected. Bone marrow biopsy (smear and core biopsy) confirmed the diagnosis (Fig. 1). JAK-2V617F, bcr-abl, CALR- and MPL- mutations turned out negative. PFA 100® test was normal, but von Willebrand factor (vWF) activity and vWF ratio were decreased, consistent with an acquired von Willebrand syndrome (vWS). Based on these results a cytoreductive treatment with hydroxycarbamide was initiated.

**Fig. 2:**
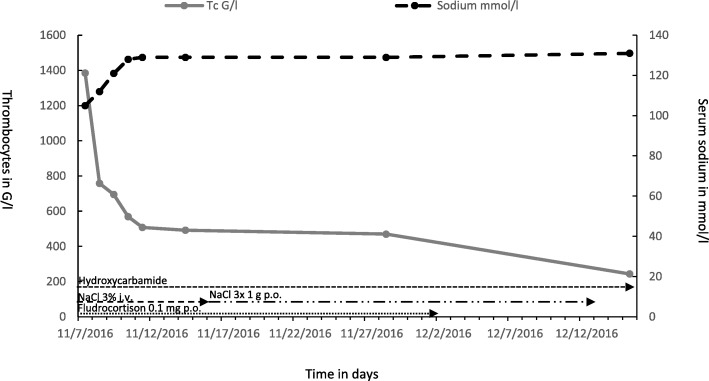
Time course of platelets and serum sodium

Because of the life-threatening degree of hyponatremia the patient was transferred to the ICU. In search of the reason for the hyponatremia, a diagnostic work up was started. After exclusion of renal failure (creatinine 33 umol/l, GFR 133 ml/min), use of diuretics, hypocortisolism and hypothyroidism, SIADH and CSW were the main differential diagnoses. Very low serum sodium (105 mmol/l) and high urinary sodium (22, later increasing up to 240 mmol/l) were consistent with both SIADH and CSW (Table 1). However, central venous pressure was low (3 mmHg) and remained low even under high saline infusion indicating persistent hypovolemia, which favored the diagnosis of CSW. Serum sodium was slowly increased with NaCl solution, initially 3% and later 0.9%. After stopping intravenous sodium, sodium level could only be maintained with supplementation of NaCl capsules and fludrocortison 0.1 mg daily, indicating ongoing salt loss. Concomitantly, the patient showed an impressive salt craving. After 3 weeks under continuous hydroxycarbamide therapy, thrombocyte counts normalized, serum sodium returned to normal and all salt substitutions could be stopped (Fig. 2). In parallel, the impaired mental condition of the patient slowly improved and finally returned to normal. The cytoreductive therapy (hydroxycarbamide 500 mg/day) was maintained.

**Table 1:** Laboratory findings at initial presentation

Parameter	Unit	Normal range	Patient	CWS	SIADH
Serum Sodium	mmol/l	[135–145]	105	< 135	low
Serum osmolality	mOms/kg	[280–300]	218	low	low
Uric Acid	μmol/l	[202–416]	95.7	low	low
Urine Sodium	mmol/l		22 *	> 20	> 20
Urine osmolality	mOms/kg		530	> 100 (300)	> 100
Hypovolemia			yes	yes	no
Leucocytes	G/l	[3.5–10]	49.7		
Hemoglobin	g/l	[140–180]	98		
Thrombocytes	g/l	[139–335]	1385		
C-reactive protein	mg/l	[< 5]	8.3		
Procalcitonin	ng/ml	[< 0.5]	0.08		
Creatinine	μmol/l	[59–104]	33		
GFR	ml/min	[> 90]	133		

* Values up to 240 mmol/l despite hypovolemia were measured in the course of disease

## Discussion and conclusion

Many complications occur in MPN, especially with very high thrombocyte counts. Due to disturbances in microcirculation, patients complain about erythromelalgia (painful redness, swelling and burning) in fingers and toes, impaired vision, dizziness and headache. Most threatening are venous or arterial thromboembolic complications as well as hemorrhages due to impaired thrombocyte function or acquired vWS. Our patient presented with almost all of these symptoms; even the abdominal pain may be interpreted due to microcirculatory disturbances. Exact classification of the MPN could not be worked out, therefore the disorder must be classified as myeloproliferative neoplasm unclassifiable (MPN-U) [6]. Other causes of myeloproliferation (chronic myelogenous leukemia, severe infection, iron deficiency) could be ruled out.

Severe hyponatremia was interpreted as a consequence of CSW/RWS, supported by high urinary sodium loss, persistent sodium loss despite low volume status and clinically exquisite salt craving [7–9]. Differentiation between CSW/RWS and SIADH is challenging, since laboratory findings may be completely overlapping - only volume status (central venous pressure, orthostatic hypotension) is helpful to distinguish between the two entities (Table 1): whereas SIADH patients suffer from volume expansion due to ADH-mediated renal water retention, CSW/RWS patients have renal sodium loss due to elevated atrial or brain natriuretic peptide. Correct diagnosis is important, since SIADH is treated by fluid restriction, whereas CSW/RWS requires sodium and fluid replacement.

The pathomechanism for CSW/RWS in our patient is speculative: ischemic microtraumata in the brain caused by microcirculatory disturbance and consecutive hypoxia may have promoted CSW/RWS in the context of a preexisting microangiopathy which was seen in the initial MRI scan of the brain. This hypothesis is supported by the prompt and complete recovery of serum sodium and mental disturbances after thrombocytes started to fall under cytoreductive therapy and finally returned into normal ranges. We cannot fully rule out a microangiopathic disorder also in the renal vessels leading to hpoxemia in the kidney as additional pathomechamism for the renal salt wasting. However, the absence of signs of acute renal failure and the concomitant neuropsychiatric symptoms clearly support a primary microangiopathic disorders in the brain. To the best of our knowledge this is the first case describing CSW/RWS as a consequence of MPN.

## Data Availability

NA

## Abbreviations

CWS: Cerebral salt wasting
MPN: Myeloproliferative Neoplasia,
RWS: Renal salt wasting
SIADH: syndrome of inappropriate antidiuretic hormone secretion
